# What’s Under the Veil?

**DOI:** 10.5826/dpc.1103a42

**Published:** 2021-07-08

**Authors:** Michela Starace, Miriam Anna Carpanese, Francesca Bruni, Iria Neri, Bianca Maria Piraccini, Aurora Alessandrini

**Affiliations:** Dermatology, Department of Experimental, Diagnostic and Specialty Medicine, University of Bologna, Italy

**Keywords:** Phthirus pubis, parasite infestation, scalp involvement, sexually transmitted disease

## Introduction

Phthirus pubis is an ectoparasite, whose only host are humans. Phthirus pubis is responsible for the corresponding parasitic infestation that is sexually transmitted and usually affects the genital areas. Despite being rare, scalp involvement is becoming increasingly frequent because of the increasing habit of pubic hair removal. We present the case of a pregnant woman with isolated scalp involvement and a diffuse cutaneous rash of the body.

## Case presentation

A 21 year old woman from Pakistan, 22 weeks pregnant, presented with a widespread intensely pruritic cutaneous rash of the body that persisted in the last 1 month. She had been previously examined at the maternity department and at the emergency room without a diagnosis.

**Figure 1:**
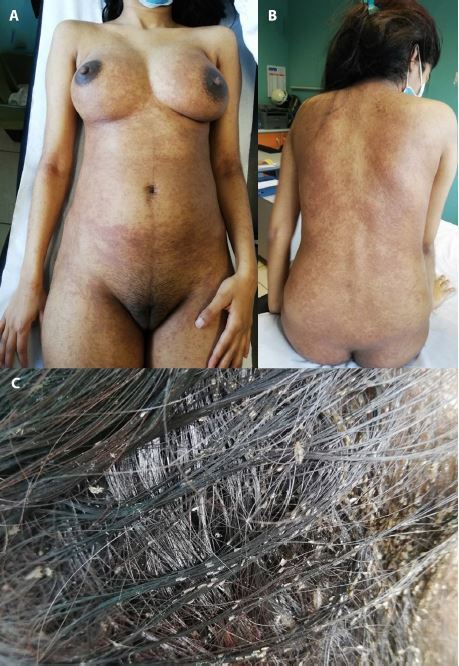
(A) Reddish and itchy papules surrounded by erythema and scratching lesions over the trunk, the legs and the arms. (B) over the back. (C) Live lice and nits covering the scalp of the patient.

Dermatological examination revealed reddish and itchy papules surrounded by erythema and scratching lesions over the trunk, the arms, and the back. (Figure 1, A and B). The Patient was asked to remove the veil from her head to facilitate the physical examination. With some resistance and difficulty, the patient finally removed the veil. This was the first time her scalp was examined as no one had ever asked her to do so in the previous examinations. The scalp was completely covered in both live lice and nits. A video of walking lice was also taken (Supplementary Material). (Figure 1C). The presence of crusty and scaly plaques over the patient’s ear and the neck indicated a secondary impetiginisation, due to scratching. No lice were found upon body inspection. When asked about her clinical history the patient confirmed that her husband also presented the same symptoms, especially at the level of the genital area, on pubic hairs. This anamnestic data and the identification of the lice allowed to diagnose a Phthirus pubis infestation that was exclusively located on the scalp.

Treatment included total hair shaving and successive application of a malathion solution.

## Conclusion

Phthirus pubis is an obligate ectoparasite, it is usually sexually transmitted and spreads via the direct contact whit an affected person. Phthirus pubis classically affects pubic, axillary and body hair, but also beard, scalp and eyelash infestation have been described. The scalp involvement is very rare, in these cases it might be the only location affected, as experienced by our patient. The increasing diffusion of pubic hair removal’s habit, destroying the lice’s natural habitat is an effective preventive procedure against Phthirus pubis [1]. Phthirus pubis showed the ability to change its tropism, leading to increasingly frequent atypical infestations in other parts of the body such as scalp, eyebrows, axillae, and trunk. As the atypical patterns of pubic lice infestation are becoming more frequent compared to the past, it is important to recognise Phthirus pubis in cases of resistant pruritus and dermatitis [2].

This reported case highlights the importance of considering an eventual Phthirus pubis infestation in patients presenting a lice infestation, exclusively affecting the scalp. This consideration allows for a correct diagnosis and treatment, including the treatment of sexual partners. Finally, another important take-home message is to always perform a full body examination, also in parts where the patient denies the disease.

## Supplementary Information
